# Delayed Traumatic Diaphragmatic Hernia Presenting With Gastric Volvulus and Necrosis: A Case Report and Literature Review

**DOI:** 10.7759/cureus.106851

**Published:** 2026-04-11

**Authors:** Fernanda D Parra Gómez, Luis G Ortega Figueroa, Wilbert M Gamboa Ríos, Carlos Luevanos Mercado, Jesús Rodríguez Cruz, Valeria Carlos Venegas, Aurelio O Cervantes Franco, Rodolfo Chávez Magallón

**Affiliations:** 1 General Surgery, Hospital General de Zona No. 33/Instituto Mexicano del Seguro Social, Bahía de Banderas, MEX

**Keywords:** delayed presentation, gastric necrosis, gastric volvulus, intrathoracic stomach, traumatic diaphragmatic hernia

## Abstract

Traumatic diaphragmatic hernia (TDH) is an uncommon condition resulting from blunt or penetrating thoracoabdominal trauma and is frequently underdiagnosed due to its nonspecific presentation. Delayed diagnosis may occur months or even years after the initial injury, increasing the risk of serious complications. Left-sided involvement is more common, and defects typically occur in areas of embryologic weakness.

The persistent pressure gradient between the abdominal and thoracic cavities promotes progressive herniation of abdominal viscera. The stomach is commonly involved, and its displacement into the thoracic cavity may predispose to gastric volvulus. This can compromise vascular perfusion, leading to ischemia, necrosis, and perforation.

We report the case of a 25-year-old male with a history of penetrating thoracoabdominal trauma due to a stab wound to the left hemithorax four years prior to presentation. He was admitted with a four-day history of progressive left upper quadrant abdominal pain associated with nausea. Imaging studies revealed a left-sided diaphragmatic hernia with intrathoracic herniation of the stomach, complicated by gastric volvulus and ischemia.

Contrast-enhanced computed tomography was essential for diagnosis, allowing precise identification of the diaphragmatic defect, herniated viscera, and associated complications, thereby guiding surgical management.

The patient underwent surgical intervention with reduction of herniated contents, partial gastrectomy due to extensive necrosis, and primary repair of the diaphragmatic defect.

This case underscores the potential for rapid progression to life-threatening complications, including gastric necrosis, in delayed TDH. Early recognition and prompt surgical intervention remain critical to improving outcomes.

## Introduction

Traumatic diaphragmatic hernia (TDH) is an uncommon but clinically significant condition resulting from blunt or penetrating thoracoabdominal trauma. Although its reported incidence is relatively low, ranging from less than 1% to 6% in blunt trauma and higher in penetrating injuries, diagnosis remains challenging due to its often nonspecific clinical presentation [1].

The diaphragm is particularly susceptible to injury at embryologic fusion points, most commonly in the posterolateral region. Left-sided hernias account for most cases, whereas right-sided defects are less frequent because of the protective effect of the liver [2]. When diaphragmatic injury is not recognized at the time of trauma, the pressure gradient between the abdominal and thoracic cavities may progressively promote visceral herniation into the chest.

Delayed presentation of TDH may occur months to decades after the initial injury. During this interval, patients may remain asymptomatic or present with intermittent respiratory or gastrointestinal symptoms. As herniation progresses, the risk of incarceration, strangulation, and ischemia increases, potentially leading to life-threatening complications [3].

The stomach is one of the most frequently herniated organs in TDH. Its abnormal intrathoracic position predisposes it to volvulus (defined as an abnormal rotation of the stomach that may compromise blood flow, which is a rare but potentially life-threatening condition), which may compromise vascular perfusion and progress to ischemia, necrosis, and perforation if not promptly treated [1]. Cross-sectional imaging, particularly contrast-enhanced computed tomography, has become the cornerstone of diagnosis, enabling precise identification of diaphragmatic defects, herniated viscera, and associated complications [4].

Surgical intervention remains the cornerstone of treatment, particularly in symptomatic or complicated cases. An abdominal approach is generally preferred in acute settings because it allows reduction of herniated contents, assessment of organ viability, and repair of the diaphragmatic defect. In cases of gastric necrosis, resection of nonviable tissue and reconstruction may be required [2].

We present a case of delayed TDH complicated by gastric volvulus and necrosis, highlighting the diagnostic and surgical challenges associated with this rare but severe condition.

## Case presentation

A 25-year-old male with a history of penetrating thoracoabdominal trauma secondary to a stab wound to the left hemithorax four years earlier presented with a four-day history of progressively worsening left upper quadrant abdominal pain, accompanied by nausea. These symptoms raised initial concern for an intra-abdominal process.

On admission, the patient was afebrile but tachycardic and tachypneic. Physical examination revealed no signs of peritoneal irritation. Initial laboratory studies showed a white blood cell count of 9.6 × 10³/µL, hemoglobin of 14.2 g/dL, and platelet count of 368 × 10³/µL (Table 1).

**Table 1:** Laboratory findings at initial presentation.

Test	Value	Normal Range	Interpretation
Hemoglobin (Hb)	14.2 g/dL	12-17 g/dL	Within normal range
White blood cell count (WBC)	9.6 × 10³/µL	5-10 × 10³/µL	Within normal range
Platelet count	368 × 10³/µL	150-400 × 10³/µL	Within normal range

**Figure 1:**
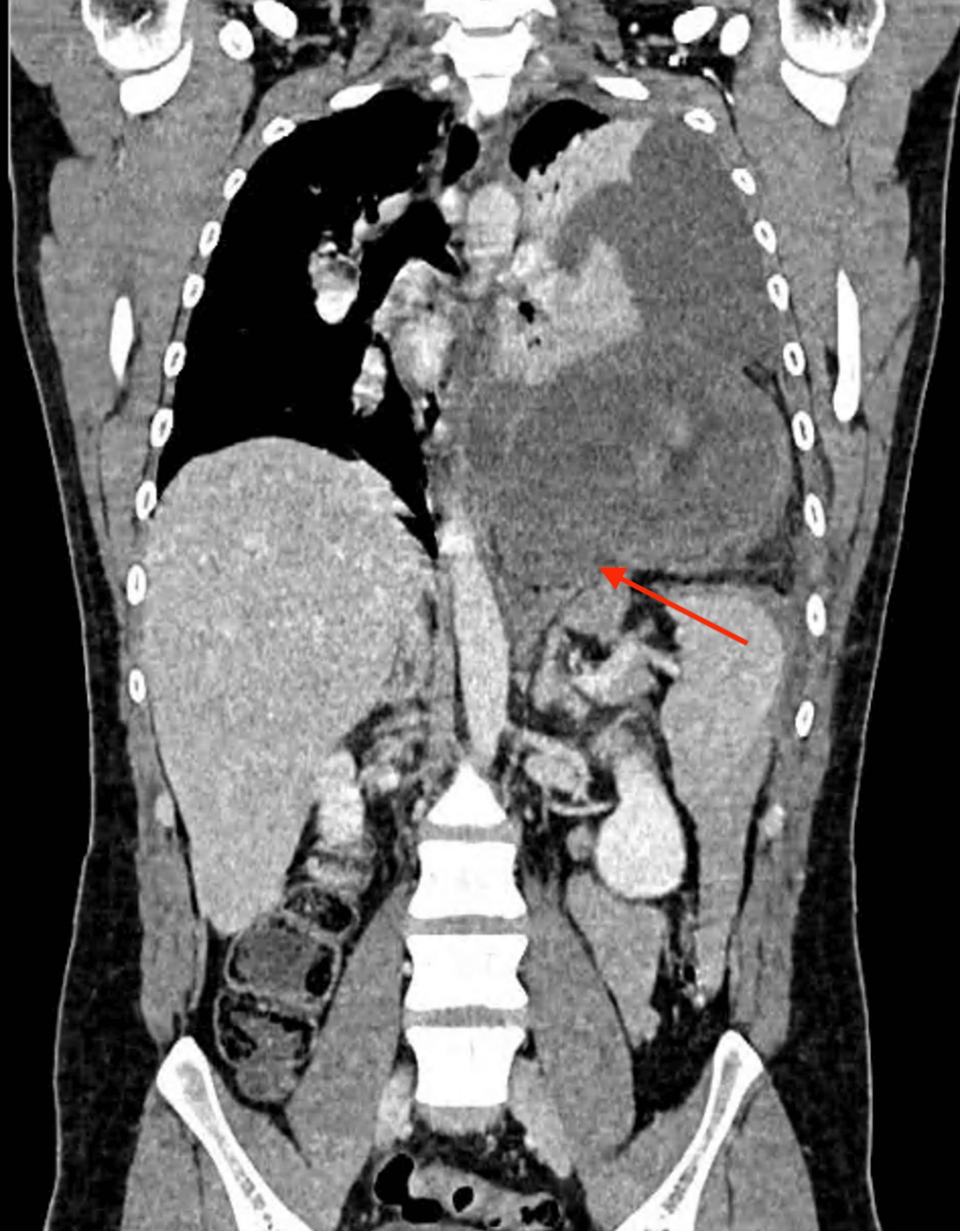
Coronal contrast-enhanced CT scan demonstrating a left-sided diaphragmatic defect (arrow) with herniation of the stomach into the thoracic cavity, associated with lung compression.

Contrast-enhanced computed tomography of the chest, abdomen, and pelvis demonstrated a left-sided diaphragmatic defect with herniation of the gastric fundus, body, and part of the antrum into the thoracic cavity, along with omental fat (Figure 1). Additional findings included left lung collapse and ipsilateral pleural effusion. These findings were consistent with a left-sided diaphragmatic hernia complicated by gastric herniation and obstruction.

During hospitalization, the patient exhibited progressive clinical deterioration, characterized by persistent nausea, clinical signs suggestive of ileus, and worsening respiratory status. A nasogastric tube was inserted, with an initial output of 400 mL. Laboratory evaluation revealed marked leukocytosis (25.3 × 10³/µL) (Table 2), raising concern for an evolving inflammatory or septic process. This deterioration also raised suspicion of underlying ischemic compromise.

**Table 2:** Follow-up laboratory findings.

Test	Value	Normal Range	Interpretation
Hemoglobin (Hb)	12.3 g/dL	12-17 g/dL	Within normal range
White blood cell count (WBC)	25.3 × 10³/µL	5-10 × 10³/µL	Marked leukocytosis
Platelet count	308 × 10³/µL	150-400 × 10³/µL	Within normal range

Given the patient’s clinical deterioration, surgical intervention was undertaken. An initial laparoscopic approach was attempted but converted to an open exploratory laparotomy due to intraoperative findings, 48 hours after admission.

**Figure 2:**
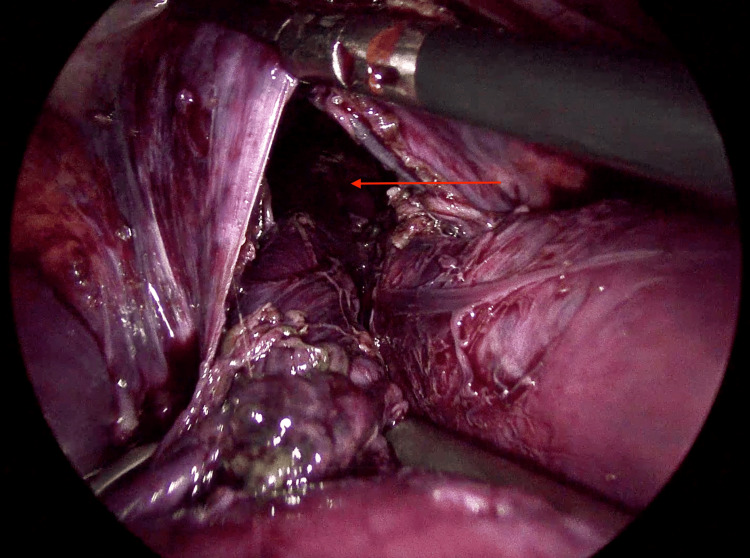
Intraoperative laparoscopic view demonstrating a diaphragmatic defect measuring approximately 2 cm (arrow), with herniation of abdominal contents into the thoracic cavity.

**Figure 3:**
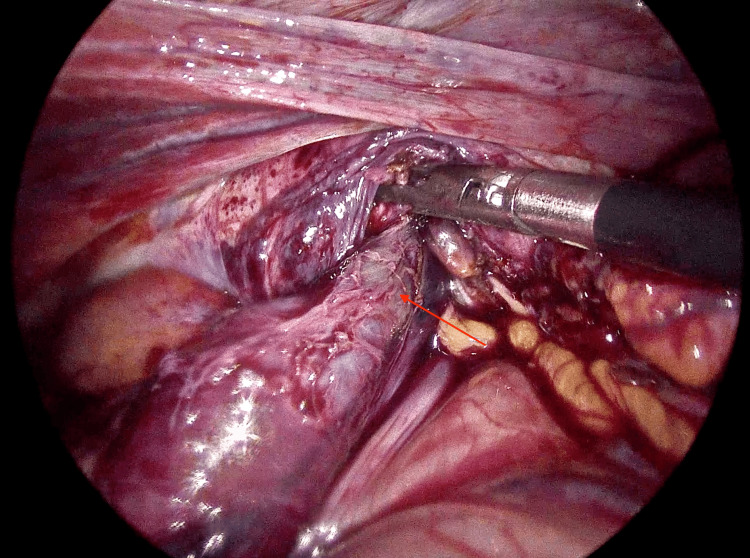
Herniated stomach through the diaphragmatic defect (arrow), consistent with a complicated diaphragmatic hernia associated with mesenteroaxial gastric volvulus.

**Figure 4:**
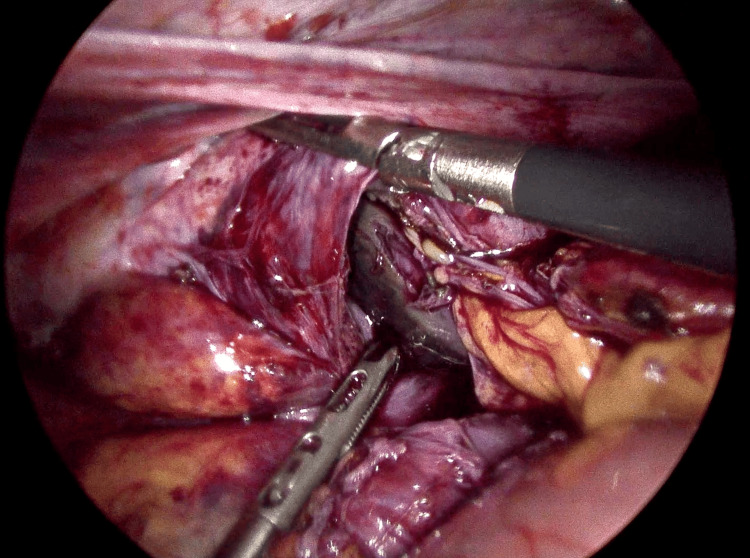
Extensive gastric ischemia with areas of necrosis involving the stomach, evidenced by dark discoloration and loss of normal serosal appearance.

Intraoperatively, a diaphragmatic defect measuring approximately 2 cm was identified, associated with a complicated diaphragmatic hernia and mesenteroaxial gastric volvulus (Figure 2). There was extensive gastric ischemia and necrosis, with multiple perforations involving the cardia, fundus, and body of the stomach (Figures 3-4). Approximately 1200 mL of purulent intra-abdominal fluid was encountered within the abdominal cavity. A concomitant splenic capsular laceration was also noted.

A proximal partial gastrectomy was performed, followed by reduction of herniated contents and primary repair of the diaphragmatic defect. A left-sided chest tube and a central venous catheter were placed.

Postoperatively, the patient was admitted to the intensive care unit under sedation and developed septic shock requiring initiation of vasopressor and supportive therapy. Despite initial stabilization, his clinical course was complicated by progressive respiratory failure and a persistent systemic inflammatory response.

A second surgical intervention was performed 48 hours later, consisting of exploratory laparotomy, esophagogastric reconstruction, placement of a feeding jejunostomy, and peritoneal lavage. Intraoperative findings included dense adhesions and a minor aortic laceration without significant hemodynamic compromise.

Despite aggressive multidisciplinary management, the patient developed multiple organ dysfunction syndrome and died on hospital day eight.

## Discussion

Delayed TDH represents a frequently missed diagnosis in penetrating thoracoabdominal trauma, often presenting years after the initial injury with life-threatening complications. In our case, the four-year interval between trauma and presentation highlights the potential for long asymptomatic periods followed by abrupt clinical deterioration [1,3].

Our case illustrates the clinical consequences of missed diaphragmatic injuries, particularly the progression from a latent phase to a complication phase characterized by visceral incarceration, strangulation, and ischemia. This progression ultimately resulted in gastric volvulus with necrosis and perforation.

The stomach is among the most frequently herniated organs in TDH. Its intrathoracic displacement alters normal anatomy and predisposes it to volvulus. Mesenteroaxial gastric volvulus, as observed in our patient, may rapidly compromise vascular perfusion, leading to ischemia and necrosis if not promptly treated [2,5].

Computed tomography remains the imaging modality of choice, allowing accurate identification of diaphragmatic defects, herniated viscera, and associated complications. Specific radiologic signs, such as the “dependent viscera” sign, are highly suggestive of diaphragmatic rupture and may facilitate early diagnosis in equivocal cases [4].

Recent reports have further demonstrated the heterogeneous and often insidious presentation of diaphragmatic hernias in adults. While some patients remain asymptomatic for years, others present with acute life-threatening complications. Adult Bochdalek hernias may present with nonspecific respiratory and gastrointestinal symptoms or remain asymptomatic until incidentally detected, contributing significantly to delayed diagnosis and increased risk of complications [6]. This variability contributes to delayed diagnosis and underscores the need for a high index of clinical suspicion [6-8].

These considerations have direct implications for management strategies. Surgical repair remains the definitive treatment, particularly in complicated cases. An abdominal approach is generally preferred because it allows reduction of herniated contents, assessment of viability, and management of associated intra-abdominal pathology. In our case, extensive gastric necrosis required partial gastrectomy, reflecting the severity of delayed presentation [2].

Minimally invasive approaches have demonstrated favorable outcomes in selected patients; however, open surgery remains essential in unstable patients or when significant visceral compromise is present [5,9]. Treatment decisions should therefore be individualized based on clinical presentation and intraoperative findings.

Delayed TDH complicated by strangulation, necrosis, and perforation is associated with high morbidity and mortality, particularly in the presence of septic shock and multiple organ dysfunction syndrome. Our patient’s outcome underscores the aggressive clinical course of this condition once advanced complications develop and highlights the critical importance of early recognition and timely surgical intervention.

This case reinforces the importance of maintaining a high index of suspicion for diaphragmatic injury in patients with a history of thoracoabdominal trauma, even years after the initial event, as delayed diagnosis may lead to catastrophic complications.

Limitations

This report is limited by its single-case design, retrospective nature, and lack of long-term follow-up. These factors restrict generalizability and outcome assessment beyond the acute phase.

## Conclusions

Delayed TDH should be considered in patients with a history of thoracoabdominal trauma presenting with nonspecific thoracoabdominal symptoms, even years after the initial injury. This case underscores the potential for rapid progression to life-threatening complications, including gastric volvulus, necrosis, and multiorgan failure. These findings highlight the importance of early recognition and timely surgical intervention in similar clinical scenarios.
